# 2,4,6-Trifluoro­benzoic acid

**DOI:** 10.1107/S160053681100345X

**Published:** 2011-01-29

**Authors:** Richard Betz, Thomas Gerber

**Affiliations:** a Nelson Mandela Metropolitan University, Summerstrand Campus, Department of Chemistry, University Way, Summerstrand, PO Box 77000, Port Elizabeth 6031, South Africa

## Abstract

In the title compound, C_7_H_3_F_3_O_2_, the C—C—C angles in the ring are greater than 120° for F-bonded C atoms [123.69 (13), 123.88 (12) and 123.66 (12)°]. In the crystal, inter­molecular O—H⋯O hydrogen bonds between carboxyl groups give rise to the formation of a centrosymmetric dimer, while dispersive F⋯O contacts [2.8849 (16) Å] connect the dimers into infinite strands along the *a* axis.

## Related literature

For the crystal structure of benzoic acid (applying neutron diffraction), see: Wilson *et al.* (1996 ▶) and of *ortho*-fluoro­benzoic acid, see: Krausse & Dunken (1966 ▶). For the graph-set analysis of hydrogen bonds, see: Etter *et al.* (1990 ▶); Bernstein *et al.* (1995 ▶).
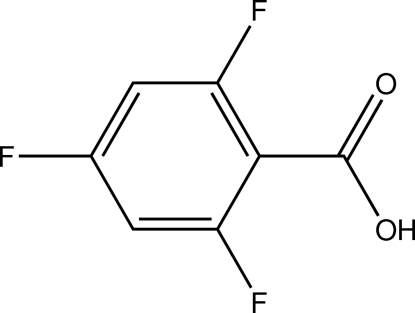


## Experimental

### 

#### Crystal data

C_7_H_3_F_3_O_2_
*M* *_r_* = 176.09
Monoclinic, 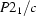

*a* = 7.2769 (3) Å
*b* = 13.7998 (6) Å
*c* = 7.3097 (3) Å
β = 115.041 (2)°
*V* = 665.04 (5) Å^3^
*Z* = 4
Mo *K*α radiation
μ = 0.18 mm^−1^
*T* = 200 K
0.59 × 0.29 × 0.18 mm

#### Data collection

Bruker APEXII CCD diffractometer
6435 measured reflections
1643 independent reflections
1394 reflections with *I* > 2σ(*I*)
*R*_int_ = 0.034

#### Refinement

*R*[*F* ^2^ > 2σ(*F* ^2^)] = 0.038
*wR*(*F* ^2^) = 0.106
*S* = 1.05
1643 reflections
111 parameters
H-atom parameters constrained
Δρ_max_ = 0.34 e Å^−3^
Δρ_min_ = −0.24 e Å^−3^

### 

Data collection: *APEX2* (Bruker, 2010 ▶); cell refinement: *SAINT* (Bruker, 2010 ▶); data reduction: *SAINT*; program(s) used to solve structure: *SHELXS97* (Sheldrick, 2008 ▶); program(s) used to refine structure: *SHELXL97* (Sheldrick, 2008 ▶); molecular graphics: *ORTEPIII* (Farrugia, 1997 ▶) and *Mercury* (Macrae *et al.*, 2006 ▶); software used to prepare material for publication: *SHELXL97* and *PLATON* (Spek, 2009 ▶).

## Supplementary Material

Crystal structure: contains datablocks I, global. DOI: 10.1107/S160053681100345X/kp2305sup1.cif

Structure factors: contains datablocks I. DOI: 10.1107/S160053681100345X/kp2305Isup2.hkl

Additional supplementary materials:  crystallographic information; 3D view; checkCIF report

## Figures and Tables

**Table 1:** Hydrogen-bond geometry (Å, °)

*D*—H⋯*A*	*D*—H	H⋯*A*	*D*⋯*A*	*D*—H⋯*A*
O1—H1⋯O2^i^	0.84	1.83	2.6560 (14)	169

Symmetry code: (i) 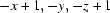.

**Fig. 1.**
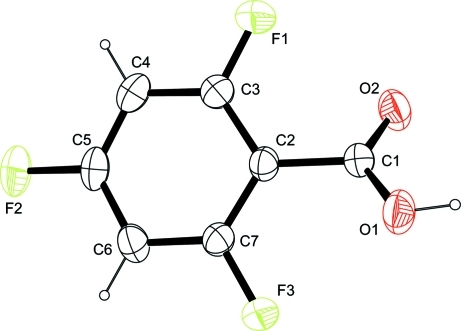
The molecular structure of the title compound, with atom labels and anisotropic displacement ellipsoids (drawn at the 50% probability level).

**Fig. 2.**
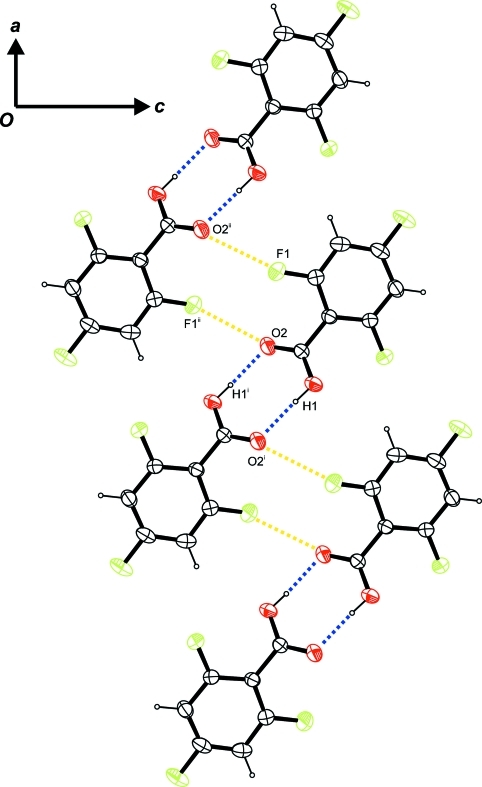
Hydrogen bonds (blue) and intermolecular F···O contacts (yellow), viewed along [0 0 1]. Symmetry operators: i 1 - x, -y, 1 - z; ii 2 - x, -y, 1 - z.

**Fig. 3.**
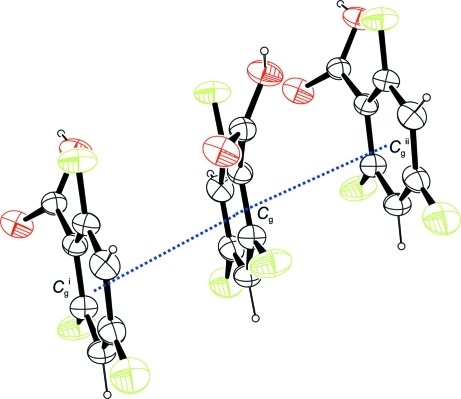
Cg···Cg distance (blue), viewed approximately along [0 1 0]. Symmetry operators: ix, 1/2 - y, -1/2 + z; iix, 1/2 - y, 1/2 + z.

**Fig. 4.**
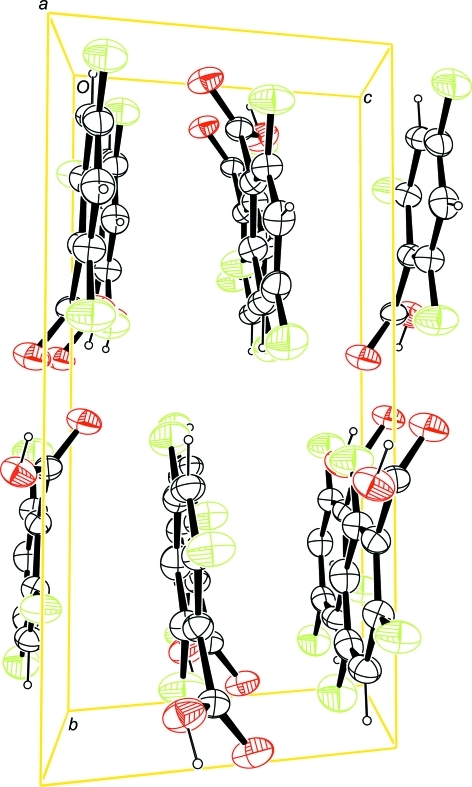
Molecular packing of the title compound, viewed approximately along [-1 0 0] (anisotropic displacement ellipsoids drawn at the 50% probability level).

## supplementary crystallographic information

### Comment

Benzoic acid has found widespread use as a ligand in coordination chemistry for a variety of transition metals and elements from the s- and p-block of the periodic system of the elements. It can act as a neutral or – upon deprotonation – an anionic ligand and serve as mono- or bidentate ligand. By varying the substituents on the phenyl moiety, the acidity of the carboxyl group can be fine-tuned. At the beginning of a comprehensive study aimed at rationalizing the coordination behaviour of various benzoic acid derivatives towards a number of transition metals in dependence of the *p*H value of the reaction batches it seemed interesting to determine the crystal structure of the title compound to enable comparative studies.

The C–C–C angles in the phenyl ring are found to be invariably larger than 120° for C-atoms bonded to F-atoms while the remaining C–C–C angles are measured at values smaller than 120°. The biggest deviation is found for the C-atom bearing the carboxyl group where a value of only about 115° is detected. The least-squares plane defined by the atoms of the carboxyl group encloses an angle of 38.17 (7)° with the plane of the aromatic system (Fig. 1).

In the crystal structure, intermolecular hydrogen bonds connect two molecules to centrosymmtric dimeric units. These dimers are joined by dispersive F···O contacts to infinite strands along the crystallographic *a* axis. In terms of graph-set analysis, the unitary descriptor for the hydrogen bonds is *R*^2^_2_(8) while the F···O contacts are described by a *R*^2^_2_(10) descriptor on the unitary level (Fig. 2).

The aromatic rings of the title compound show π-stacking with the COOH group rotated by about 90° with respect to the carboxyl groups of two neighbouring molecules (Fig. 3). The distance between two centers of gravity was determined to be 3.7501 (8) Å, the distance between the perpendicularily- projected centers of gravity of two neighbouring aromatic moieties with respect to the carbocycles was found to be 3.5507 (5) Å and 3.4651 (5) Å, respectively. The molecular packing of the compound is shown in Figure 4.

### Experimental

The compound was obtained commercially from Fluorochem. Crystals suitable for X-ray diffraction were obtained upon slow evaporation of an aqueous solution of the compound at room temperature.

### Refinement

Carbon-bound H-atoms were placed in calculated positions (C—H 0.95 Å) and were included in the refinement in the riding model approximation, with *U*(H) set to 1.2*U*_eq_(C). The H atom of the carboxylic acid group was allowed to rotate with a fixed angle around the C—O bond to best fit the experimental electron density (HFIX 147 in the *SHELX* program suite (Sheldrick, 2008)).

### Crystal data

**Table:**

C_7_H_3_F_3_O_2_	*F*(000) = 352
*M**_r_* = 176.09	*D*_x_ = 1.759 Mg m^−^^3^
Monoclinic, *P*2_1_/*c*	Mo *K*α radiation, λ = 0.71073 Å
Hall symbol: -P 2ybc	Cell parameters from 4021 reflections
*a* = 7.2769 (3) Å	θ = 3.0–28.3°
*b* = 13.7998 (6) Å	µ = 0.18 mm^−^^1^
*c* = 7.3097 (3) Å	*T* = 200 K
β = 115.041 (2)°	Platelet, colourless
*V* = 665.04 (5) Å^3^	0.59 × 0.29 × 0.18 mm
*Z* = 4	

### Data collection

**Table:**

Bruker APEXII CCD diffractometer	1394 reflections with *I* > 2σ(*I*)
Radiation source: fine-focus sealed tube	*R*_int_ = 0.034
graphite	θ_max_ = 28.4°, θ_min_ = 3.0°
φ and ω scans	*h* = −9→9
6435 measured reflections	*k* = −18→18
1643 independent reflections	*l* = −5→9

### Refinement

**Table:**

Refinement on *F*^2^	Primary atom site location: structure-invariant direct methods
Least-squares matrix: full	Secondary atom site location: difference Fourier map
*R*[*F*^2^ > 2σ(*F*^2^)] = 0.038	Hydrogen site location: inferred from neighbouring sites
*wR*(*F*^2^) = 0.106	H-atom parameters constrained
*S* = 1.05	*w* = 1/[σ^2^(*F*_o_^2^) + (0.0562*P*)^2^ + 0.2027*P*] where *P* = (*F*_o_^2^ + 2*F*_c_^2^)/3
1643 reflections	(Δ/σ)_max_ < 0.001
111 parameters	Δρ_max_ = 0.34 e Å^−^^3^
0 restraints	Δρ_min_ = −0.24 e Å^−^^3^

### Fractional atomic coordinates and isotropic or equivalent isotropic displacement parameters (Å^2^)

**Table:**

	*x*	*y*	*z*	*U*_iso_*/*U*_eq_	
F1	1.07313 (14)	0.09987 (7)	0.62543 (18)	0.0511 (3)	
F2	1.17560 (15)	0.43353 (7)	0.66407 (16)	0.0529 (3)	
F3	0.53852 (12)	0.31106 (6)	0.54957 (15)	0.0430 (3)	
O1	0.52914 (16)	0.11612 (8)	0.61602 (17)	0.0400 (3)	
H1	0.4596	0.0652	0.5829	0.060*	
O2	0.67873 (17)	0.04775 (7)	0.43736 (17)	0.0409 (3)	
C1	0.65846 (19)	0.11507 (9)	0.5422 (2)	0.0286 (3)	
C2	0.79605 (19)	0.20020 (9)	0.58194 (19)	0.0278 (3)	
C3	0.9984 (2)	0.18927 (10)	0.6151 (2)	0.0322 (3)	
C4	1.1295 (2)	0.26580 (11)	0.6441 (2)	0.0364 (3)	
H4	1.2666	0.2560	0.6663	0.044*	
C5	1.0518 (2)	0.35709 (10)	0.6392 (2)	0.0353 (3)	
C6	0.8555 (2)	0.37499 (10)	0.6104 (2)	0.0347 (3)	
H6	0.8072	0.4390	0.6095	0.043 (5)*	
C7	0.7322 (2)	0.29517 (9)	0.5829 (2)	0.0296 (3)	

### Atomic displacement parameters (Å^2^)

**Table:**

	*U*^11^	*U*^22^	*U*^33^	*U*^12^	*U*^13^	*U*^23^
F1	0.0372 (5)	0.0359 (5)	0.0811 (7)	0.0074 (4)	0.0260 (5)	0.0007 (5)
F2	0.0478 (5)	0.0459 (6)	0.0644 (7)	−0.0255 (4)	0.0233 (5)	−0.0047 (4)
F3	0.0322 (4)	0.0340 (4)	0.0686 (6)	0.0011 (3)	0.0270 (4)	−0.0033 (4)
O1	0.0402 (6)	0.0350 (5)	0.0549 (7)	−0.0126 (4)	0.0300 (5)	−0.0086 (5)
O2	0.0483 (6)	0.0286 (5)	0.0548 (7)	−0.0083 (4)	0.0306 (5)	−0.0094 (4)
C1	0.0281 (6)	0.0249 (6)	0.0337 (7)	−0.0024 (5)	0.0140 (5)	0.0006 (5)
C2	0.0276 (6)	0.0268 (6)	0.0309 (6)	−0.0043 (4)	0.0144 (5)	−0.0015 (5)
C3	0.0296 (6)	0.0312 (6)	0.0374 (7)	−0.0004 (5)	0.0157 (5)	−0.0006 (5)
C4	0.0263 (6)	0.0457 (8)	0.0377 (7)	−0.0069 (5)	0.0142 (5)	−0.0010 (6)
C5	0.0364 (7)	0.0368 (7)	0.0333 (7)	−0.0157 (5)	0.0154 (6)	−0.0035 (5)
C6	0.0404 (7)	0.0266 (6)	0.0395 (7)	−0.0061 (5)	0.0193 (6)	−0.0032 (5)
C7	0.0279 (6)	0.0296 (6)	0.0347 (7)	−0.0024 (5)	0.0167 (5)	−0.0021 (5)

### Geometric parameters (Å, °)

**Table:**

F1—C3	1.3374 (16)	C2—C3	1.3953 (17)
F2—C5	1.3486 (15)	C3—C4	1.3782 (19)
F3—C7	1.3428 (15)	C4—C5	1.375 (2)
O1—C1	1.2674 (16)	C4—H4	0.9500
O1—H1	0.8400	C5—C6	1.375 (2)
O2—C1	1.2523 (16)	C6—C7	1.3806 (18)
C1—C2	1.4903 (17)	C6—H6	0.9500
C2—C7	1.3915 (18)		
			
?···?	?		
			
C1—O1—H1	109.5	C5—C4—H4	121.7
O2—C1—O1	124.75 (12)	C3—C4—H4	121.7
O2—C1—C2	117.42 (11)	F2—C5—C4	117.98 (13)
O1—C1—C2	117.82 (11)	F2—C5—C6	118.13 (13)
C7—C2—C3	115.53 (11)	C4—C5—C6	123.88 (12)
C7—C2—C1	123.04 (11)	C5—C6—C7	116.61 (13)
C3—C2—C1	121.40 (12)	C5—C6—H6	121.7
F1—C3—C4	117.35 (12)	C7—C6—H6	121.7
F1—C3—C2	118.93 (12)	F3—C7—C6	117.62 (12)
C4—C3—C2	123.69 (13)	F3—C7—C2	118.69 (11)
C5—C4—C3	116.60 (13)	C6—C7—C2	123.66 (12)
			
O2—C1—C2—C7	140.81 (14)	C3—C4—C5—F2	−179.03 (12)
O1—C1—C2—C7	−38.89 (19)	C3—C4—C5—C6	1.0 (2)
O2—C1—C2—C3	−37.27 (19)	F2—C5—C6—C7	179.21 (13)
O1—C1—C2—C3	143.02 (14)	C4—C5—C6—C7	−0.8 (2)
C7—C2—C3—F1	177.00 (12)	C5—C6—C7—F3	−178.40 (12)
C1—C2—C3—F1	−4.8 (2)	C5—C6—C7—C2	−0.4 (2)
C7—C2—C3—C4	−1.0 (2)	C3—C2—C7—F3	179.23 (12)
C1—C2—C3—C4	177.20 (13)	C1—C2—C7—F3	1.0 (2)
F1—C3—C4—C5	−178.06 (13)	C3—C2—C7—C6	1.2 (2)
C2—C3—C4—C5	0.0 (2)	C1—C2—C7—C6	−176.97 (13)

### Hydrogen-bond geometry (Å, °)

**Table:**

*D*—H···*A*	*D*—H	H···*A*	*D*···*A*	*D*—H···*A*
O1—H1···O2^i^	0.84	1.83	2.6560 (14)	169

Symmetry codes: (i) −*x*+1, −*y*, −*z*+1.
